# Boveri’s Reaction in Meningitis

**Published:** 1918-11

**Authors:** 


**Boveri's Reaction in Meningitis.** *The Practitioner*, August, 1918.

This reaction is obtained by adding to 1 cc. of cerebro-spinal fluid an equal amount of a 1 o/o solution of permanganate of potash. If the fluid is pathological it becomes yellow; if it is normal, the violet color remains unchanged. The reaction is negative if it is delayed more than 10 minutes. The intensity and the quickness of the reaction are proportional to the amount of albumen contained in the cerebro-spinal fluid. An amount of 3 o/o of albumen, produces the reaction in a few seconds, while one of 0.40 to 0.15 requires 10 minutes.

